# Research Trends in Class II Malocclusion Treatment in Growing Individuals: A Bibliometric Study

**DOI:** 10.1016/j.identj.2024.09.014

**Published:** 2024-10-11

**Authors:** Antonino Lo Giudice, Mattia Boato, Giuseppe Palazzo

**Affiliations:** Department of General Surgery and Medical-Surgical Specialties, University of Catania, Policlinico Universitario “Gaspare Rodolico - San Marco,” Catania, Italy

**Keywords:** Class II malocclusion, Functional appliance, Headgear, Orthodontics treatment, Bibliometric analysis

## Abstract

**Introduction and aims:**

This study aims to perform a bibliometric analysis of the literature on correction of Class II malocclusion in growing individuals by evaluating the evolution and current state of treatment and to predict future research hot spots and trends.

**Methods:**

Keyword queries were used to search for articles in the Web of Science Core Collection at the electronic library of University of Catania. The resulting literature data were imported into CiteSpace 6.3.R1 and VOSviewer software to analyse authorship, countries, institutions, and keywords and to perform cluster analysis.

**Results:**

A total of 843 articles were analysed. Research in this field has shown a consistent and ongoing publication trend on the addressed topic, with a significant increase in the last decade, attributed to growing interest in skeletal anchorage. The institutions in the United States, Italy, and Brazil contributed predominantly to the investigated subject area.

**Conclusions:**

Class II correction in growing individuals using functional appliances or headgears has gained widespread attention and popularity due to the potential to counteract unfavourable maxillary and/or mandibular growth patterns, improving skeletal disharmony and facial attractiveness. A specific geographic publication trend was found for functional appliances and headgears that may represent an ethical and racial bias . Both of the latter approaches are effective in reducing the overjet. However, the primary rationale for early intervention is reducing the risk of incisal trauma and bullying episodes at childhood

## Introduction

Class II malocclusion is the most prevalent sagittal skeletal discrepancy.^1^ Different skeletal characteristics can contribute to the development of a Class II malocclusion^2^: mandibular skeletal retrusion, sagittal maxillary hyperplasia, or a posterior position of the glenoid fossa.

Functional appliances (FAs), in the form of removable or fixed devices, and headgears (HGs) are the most used and studied appliances for treating Class II malocclusion in growing children. FAs are used to stimulate sagittal mandibular growth; however, it has been demonstrated that these devices can inhibit sagittal maxillary growth compared to untreated control participants.^3^ Remodelling of the glenoid fossa and dentoalveolar effects could also contribute to the correction of the malocclusion using FAs. HGs are used to primarily inhibit sagittal maxillary growth and distalize upper molars. High-pull (occipital) or low-pull (cervical) traction can be used to control vertical maxillary growth which, in turn, can influence sagittal mandibular projection.^4^

In past years, several clinical trials and systematic reviews (including meta-analysis)^5^, ^6^, ^7^ have been published on Class II correction in growing individuals. These studies represent a valuable clinical and scientific source to comprehensively understand the clinical conditions and characteristics associated with treatment success but also with complications and side effects. However, a comprehensive bibliometric study addressing the impact of publications in this field is not available in literature In this context, bibliometric analysis quantitatively evaluates data related to authors, countries, institutions, and clustered keywords, offering preliminary insights into the development and trends within a specific field.^8^ Compared with traditional descriptive reviews, bibliometric analysis offers significant advantages in rapidly identifying key information and guiding future research directions.^9^

The aim of this study is to perform a bibliometric analysis of the literature on correction of Class II malocclusion in growing individuals. This analysis elucidates the current scientific output; the collaboration network amongst authors, countries, and institutions; and the most influential journals and identify research hot spots and trends through keyword and citation analysis. The findings of the present study would implement the actual evidence-based literature and will provide references for future research into this field.

## Material and methods

### Data collection

A literature search was conducted in the Web of Science Core Collection at the University of Catania, with a time span from 1999-01-01 to 2024-03-19. A general search query was defined to retrieve studies addressing Class II correction in growing individuals. In particular, the query could include different definitions of the same appliance or different appliance designs used for correcting Class II malocclusion with the primary aim to favour mandibular growth (FA) or inhibit maxillary growth (extraoral traction): “((ALL=((Orthodontic functional appliance OR Sander OR Harlvold OR Bionator OR Andresen OR Frankel OR Twin-block OR Harvold OR Sander OR Activator OR Extraoral Traction Appliances OR Extraoral traction OR Extra-oral traction OR Extra oral traction OR Headgear OR Cervical headgear OR High-pull headgear OR Facebow OR Face-bow))) AND ALL=(Class II malocclusion))”.

### Data screening

**Fig. 1:**
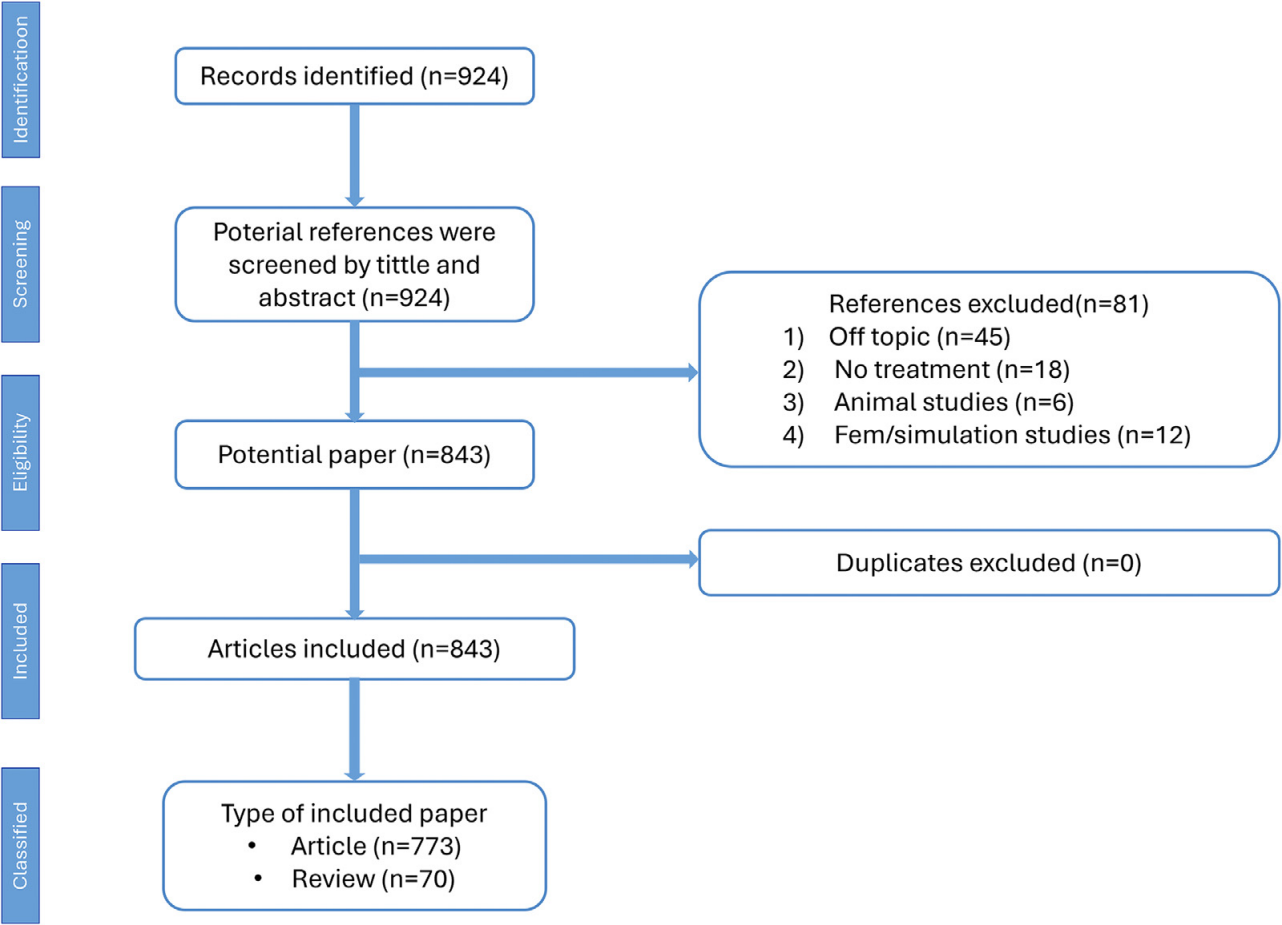
Flowchart of the study.

Two independent investigators (MB and ALG) screened the documents according to type and information integrity. The exclusion criteria involved both the methodology and the publication type of the studies. Concerning methodology, studies were excluded if they were based on (1) animal investigation, (2) fem/simulation investigation, (3) growth observation (no treatment), (4) single camouflage treatment with fixed appliances, (5) adult treatment, including orthognathic surgery, (6) related off topics. Concerning publication type, studies were excluded if they were editorial materials, letters, news, bibliographic items, conference proceedings, recensions, or reprints. Articles with incomplete information for bibliometric analysis were also excluded. Thus, the present bibliometric analysis included only articles and reviews, without language restriction (Figure 1). In case of disagreements between the 2 independent investigators, a third researcher (GP) was consulted.

### Data processing

The dataset of the included studies was imported as plain text into the CiteSpace 6.3.R1 program, and the data processing for bibliometric analysis was based on the integration of software analysis and manual analysis. CiteSpace 6.3.R1 software offered rankings and centrality measures for authors, nations, and institutions, journals and keyword clustering. CiteSpace 6.3.R1 was used to analyse authors’ contributions and collaborations as well as co-cited references. The selection threshold was lowered by “Year Slice” from 1 to 2 to meet the output requirements (network size fewer than or equal to 300) and “Time period = 1999–2024,” “Year Slice = 2,” “g-index = 10,” and “Top N% = 5” (for author, country, institution, and keyword analysis and citing journal analysis) were set consistently. Other parameters were left as default settings, and neither “Pathfinder” nor “Pruning networks” were selected. VOSviewer was used during the visualisation process and the data were collated to analyse geographic distribution, international collaborations, and co-cited journals.

### Describing indicators

The results of this study are primarily presented in quantitative and percentage formats, accompanied by visual network maps. The findings comprise cluster and burst keywords analysis, influence networks (journals), and contribution and collaboration networks (authors, countries, and institutions). The visual network maps are composed of nodes and links. Each node represents a project, with the size of the node indicating its frequency of occurrence. The links between nodes represent cooperative relationships. Three structural indicators—centrality, modularity, and silhouette—are used to assess the quality of the network. Centrality, determined by calculating the shortest paths between every pair of nodes, indicates the importance of a node within the network by reflecting how central it is within a cluster or how it serves as a bridge. Nodes with high centrality are considered significant findings in the study. Keyword clustering, which groups strongly correlated nodes, can uncover related research areas and their evolution over time. The quality of the network's cluster division is gauged by the modularity score, or Q score. A Q score greater than 0.3 denotes a significant clustering structure, and a higher value denotes a well-structured network. The Q score ranges from 0 to +1. The quality of the clustering configuration is assessed using the silhouette score (S score), which has a range of −1 to +1. A network is regarded as homogenous, reasonable, or highly reliable depending on whether its S score is greater than 0.3, 0.5, or 0.7.^10^

## Results

After data screening and processing, a total of 843 articles were included for final analysis. CiteSpace 6.3.R1 software showed that of the overall articles selected, 773 were articles and 70 were reviews.

### Analysis of annual publishing volume

**Fig. 2:**
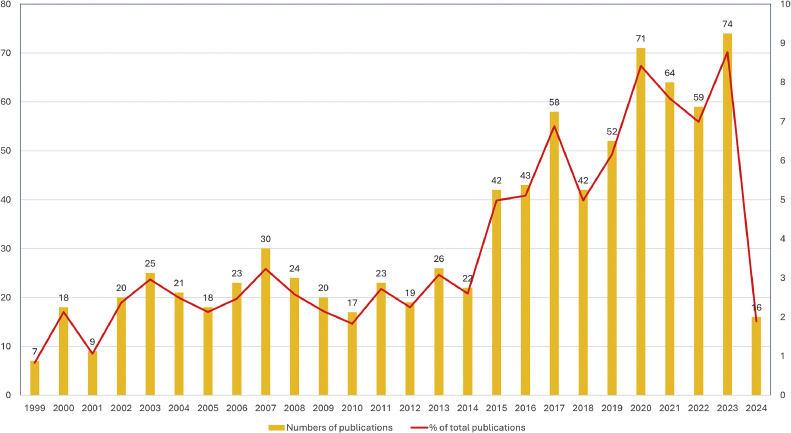
Trend for annual publication number.

The trend for annual publications number showed 2 distinct phases, with a cutoff between the years 2014 and 2015. In particular, the number of annual publications between 1999 and 2014 accounted for about 1% to 3% of the total publications retrieved (n = 843), whilst the number of annual publications between 2015 and 2023 ranged from between 5% and 9% of the total publications retrieved (Figure 2).

### Country, institution, and author analysis

**Fig. 3:**
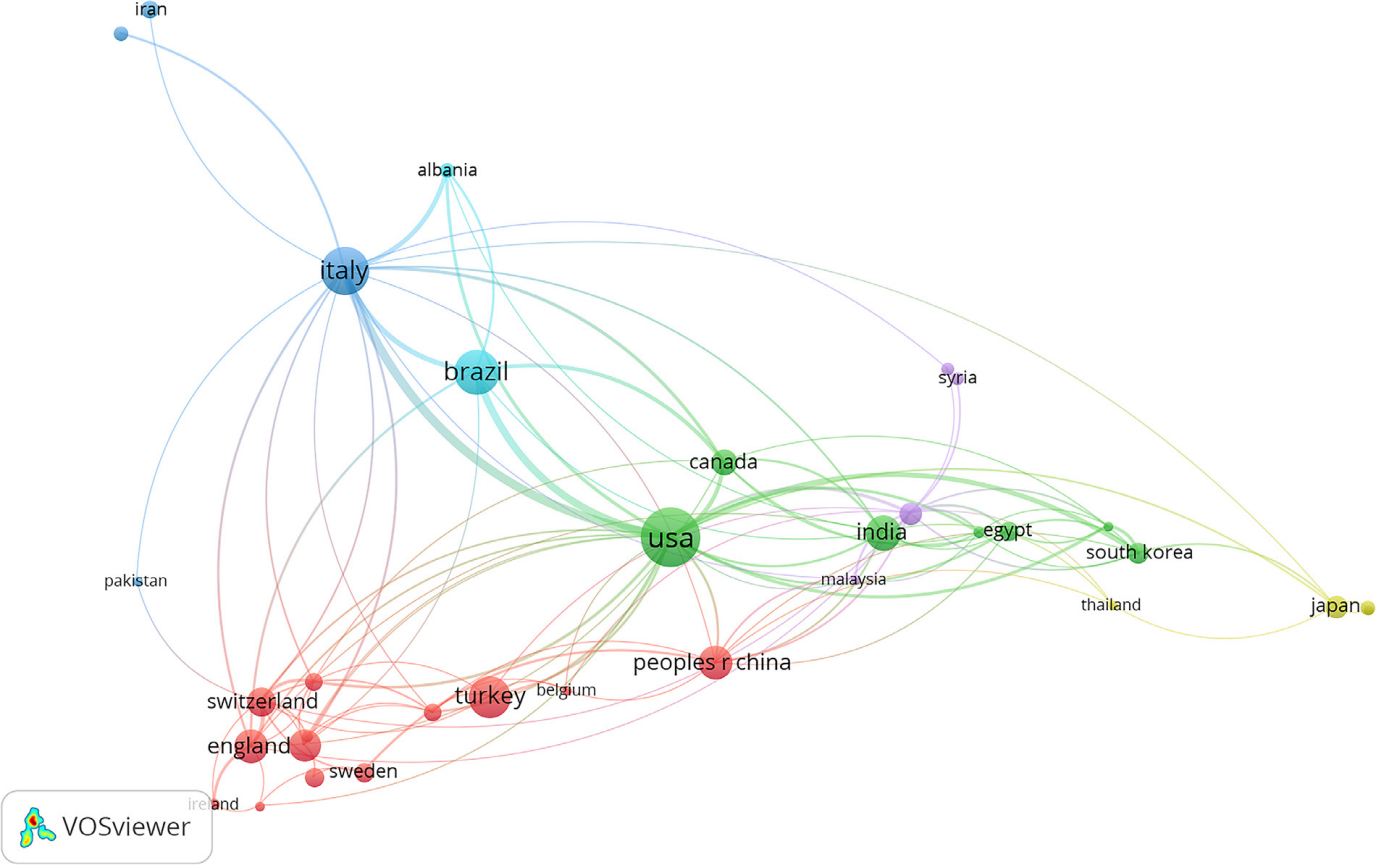
Main country co-occurrence network.

Research on the treatment of Class II malocclusion involved contributions from 66 countries. By extending the publication volume cutoff to 5, 36 countries were included in the selected period (1999–2024). Table 1 lists the 5 countries with the highest number of publications and their relative impact (percentage) on the total retrieved literature. The United States ranked first, with more than 100 publications (13.52% of the total), followed by Italy, Brazil, India, and China. There was also significant collaboration amongst different countries, with stronger participation in cooperative networks amongst the highly ranked countries (Figure 3).

**Table 1:** The 5 most productive countries.

Ranking	Country	Count	Centrality	Percentage contribution
1	US	114	0.29	13.52%
2	ITALY	89	0.29	10.55%
3	BRAZIL	28	0	3.30%
4	INDIA	20	0	2.30%
5	PEOPLES R CHINA	14	0	1.65%

**Fig. 4:**
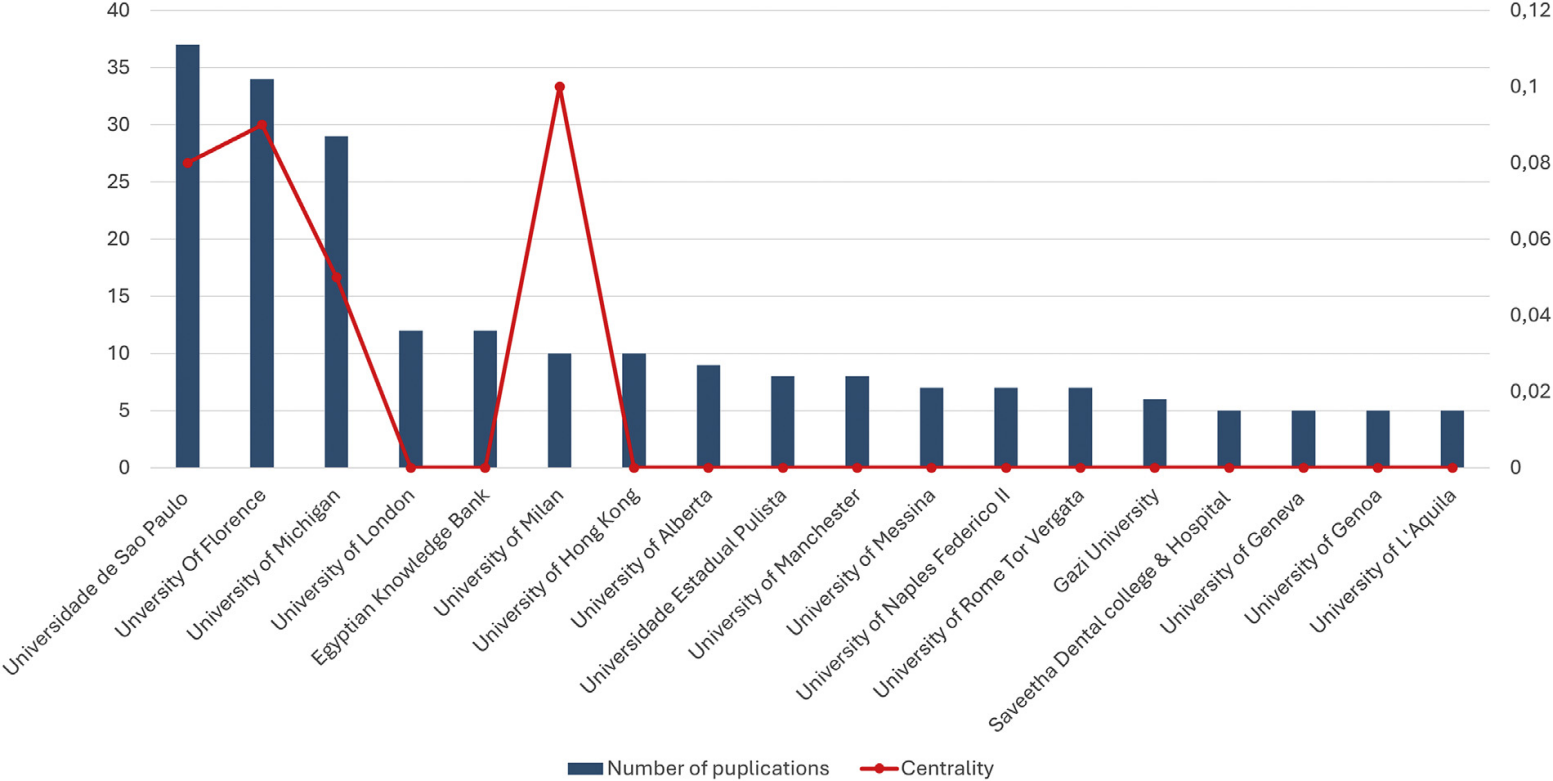
Eighteen institutions with publication volumes greater than 5.

**Fig. 5:**
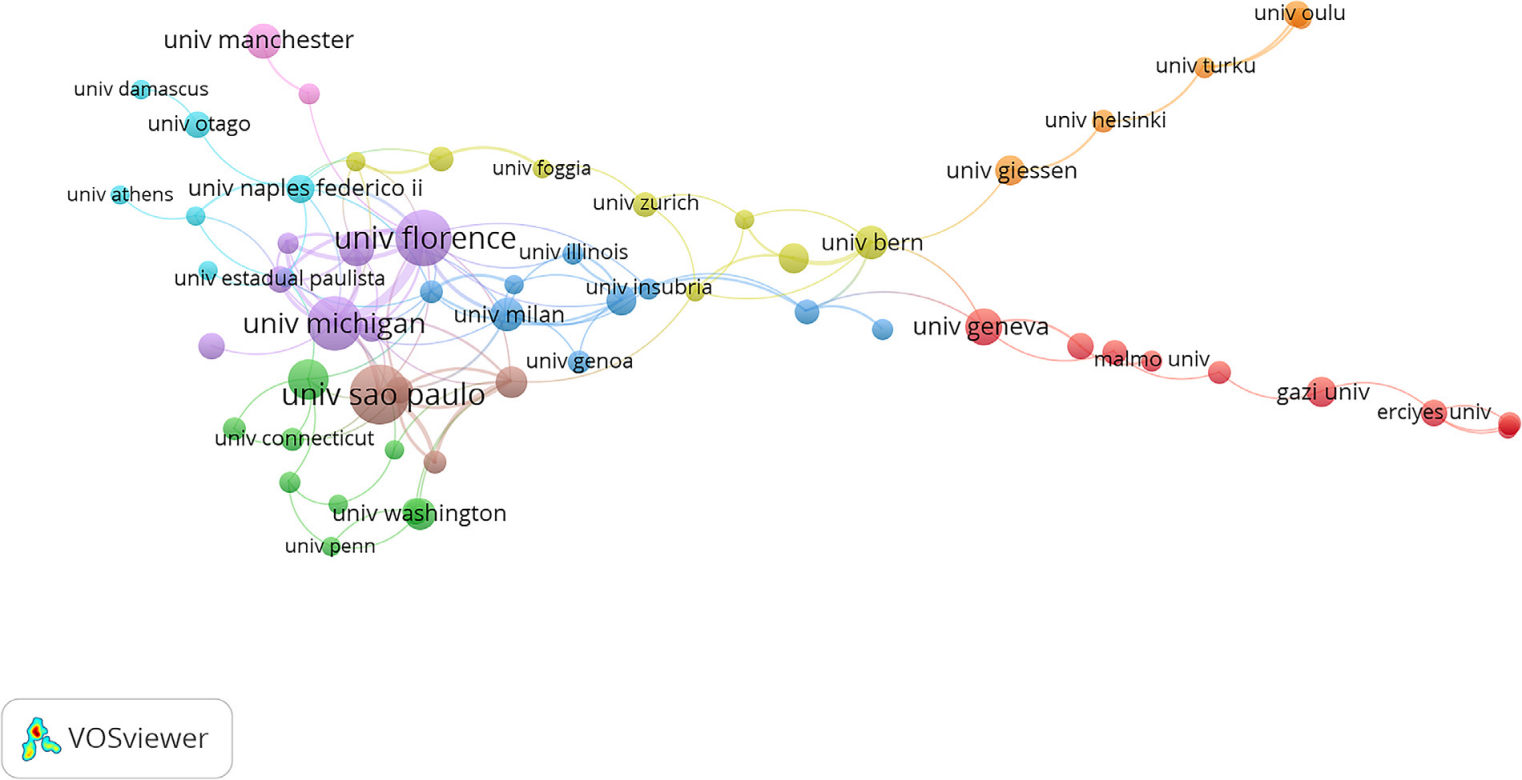
Main institution co-occurrence network.

A total of 816 institutions have contributed at least one publication to this field of research. When the threshold was set to a minimum of 5 publications, 18 institutions were identified. Italy emerged as the most represented country, with 7 institutions and the highest total number of publications (Figure 4). The University of Florence ranks first in centrality score, whilst the University of São Paulo leads in publication count. A distinct separation exists amongst the University of Florence, the University of São Paulo, and the University of Michigan compared with to other institutions, indicating their significant contributions to this research area. Figure 5 illustrates the major collaboration networks, showing that most collaborations occur amongst institutions within the same country.

**Fig. 6:**
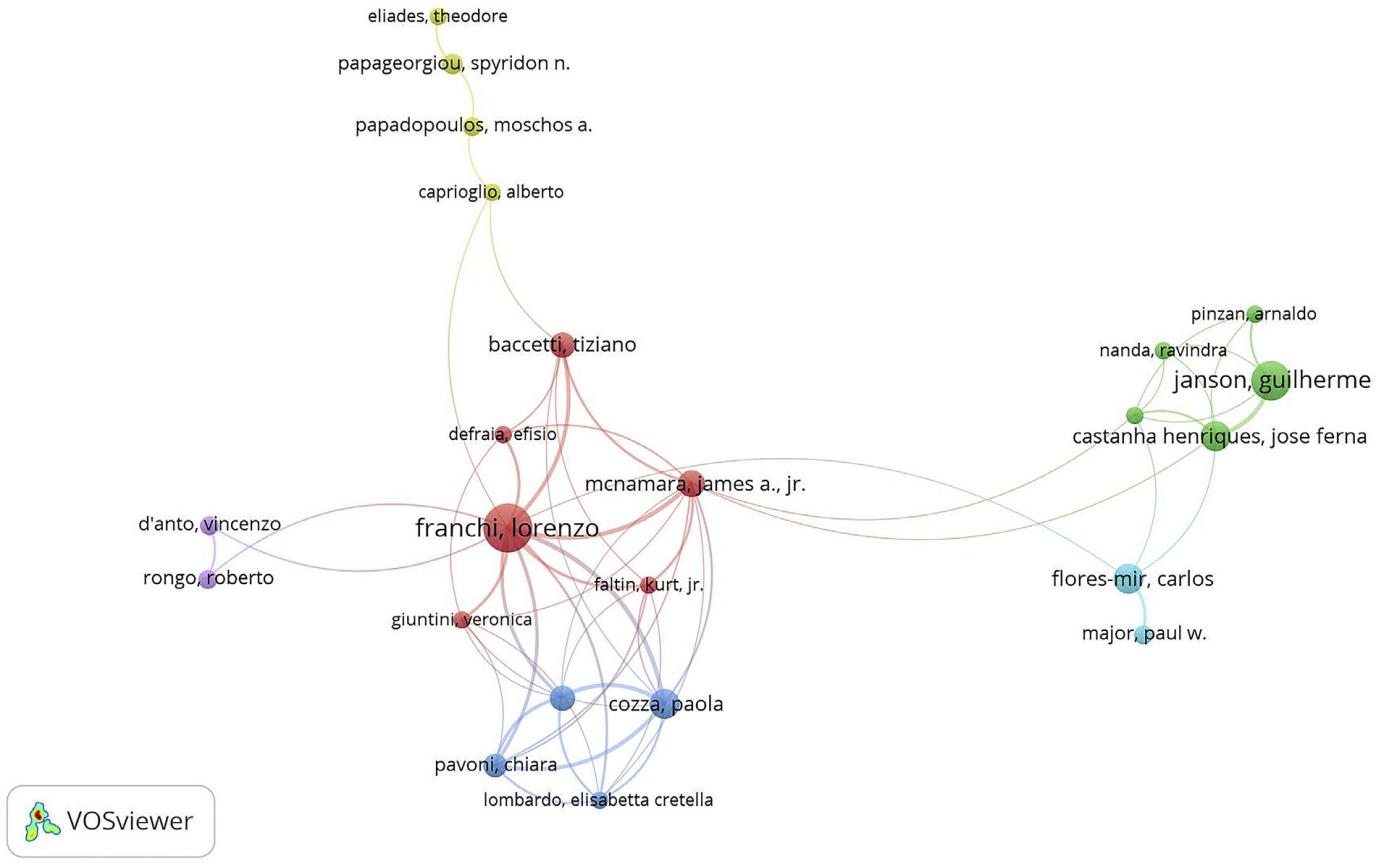
Main author co-occurrence network.

The full names and abbreviations of the same authors were consolidated into single author entries. Table 2 lists the 9 most productive authors, along with their publication counts, citation counts, and centrality scores. Of these 9 authors, only 2 have centrality scores greater than 0, indicating a higher level of extensive collaboration. Figure 6 shows major author collaboration networks, with each colour representing a network group characterised by frequent collaborations. Additionally, some collaboration is observed between different groups.

**Table 2:** The 10 most productive authors.

Ranking	Name	Count	Citations	Centrality	Country
1	Franchi, Lorenzo	33	826	0.04	Italy
2	Janson, Guilherme	21	142	0	Brazil
3	Flores-Mir, Carlos	13	297	0.01	Canada
4	Castanha Hernrique, Jose Fernando	11	80	0	Portugal
5	Cozza, Paola	11	310	0	Italy
6	McNamara, James A	10	235	0	US
7	Fleming, Padharaig S	10	188	0	England
8	lione, Roberta	9	70	0	Italy
9	Baccetti, Tiziano	8	388	0	Italy
10	Kiliaridis, Stavros	8	39	0	Greek

### Keyword analysis

**Fig. 7:**
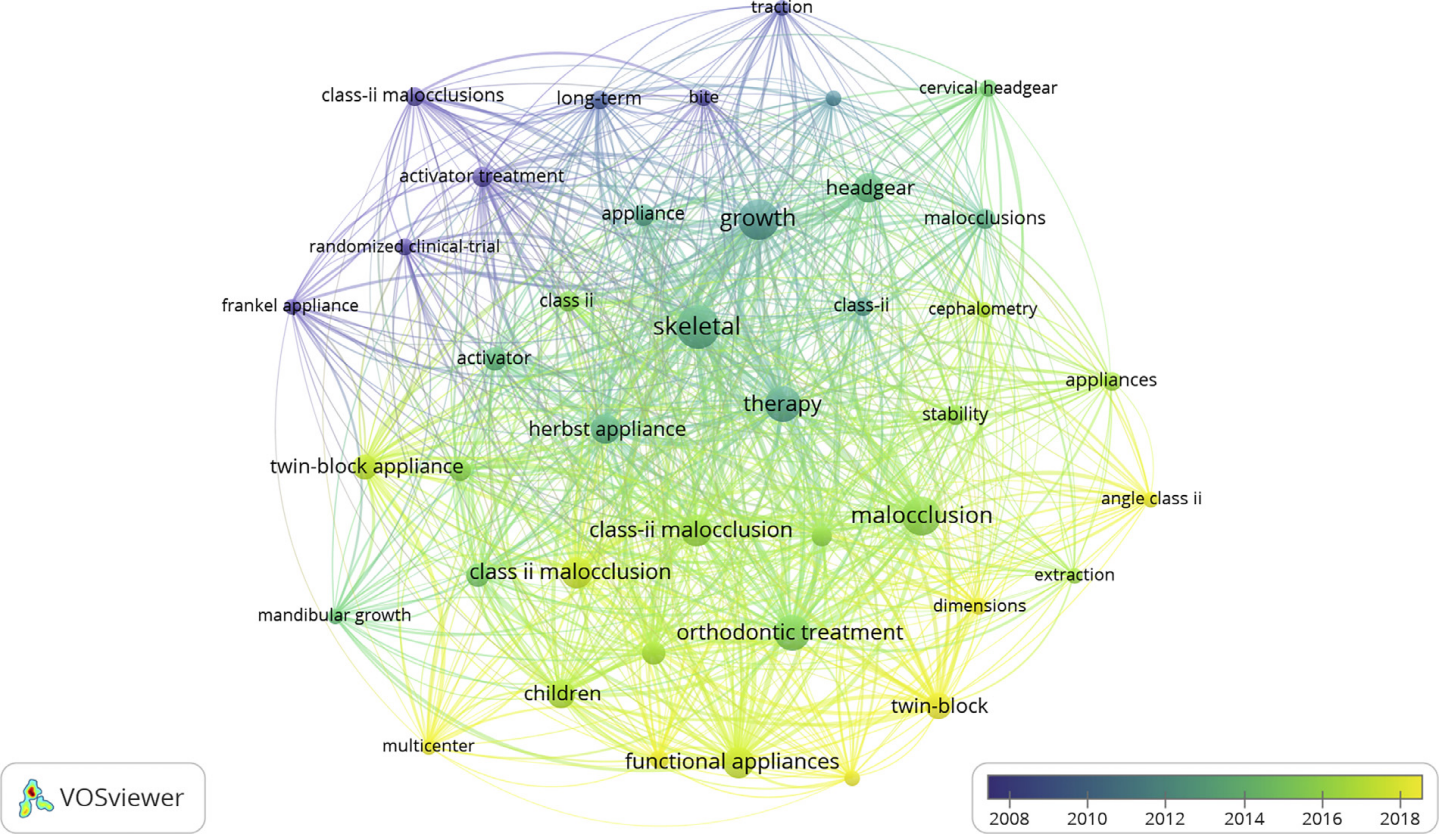
The co-occurrence of keywords that appear more than 30 times.

Keyword analysis provides information about hot spots and trends in a research field.^8^ A total of 1845 keywords were identified in the included literature, and Figure 7 displays keywords that mostly appeared in the strategy search (>30 times). The 3 most frequently occurring keywords were “skeletal,” “growth,” and “therapy,” and the top 3 keywords in terms of centrality were “growth,” “Class II malocclusion,” and “functional appliances.” These keywords are consistent with the topic addressed in the present study (Class II correction in growing individuals). When observing the keywords from top to bottom, there is consistency amongst years concerning the occurrence of FA-related keywords, whilst extraoral traction–related keywords remain located in the upper region.

### Keywords with citation burst

**Fig. 8:**
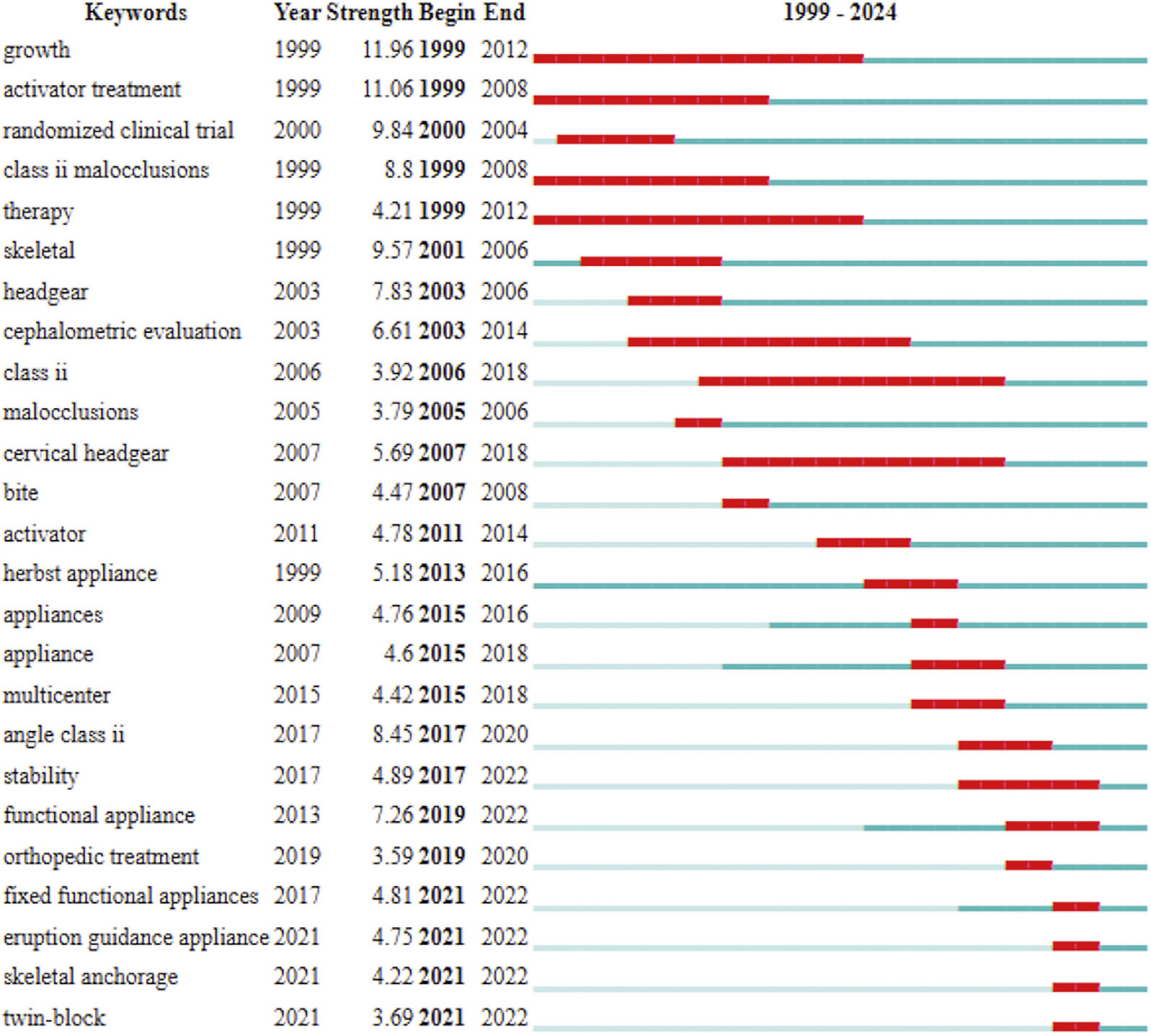
Top 25 keywords with strongest citation bursts. The red line indicates years with frequent appearances, and the green line indicates years with fewer appearances.

The burst citation maps (Figure 8) revealed 3 distinct phases based on the years when keywords began receiving high citations. Before 2011, significant research focused on growth factors, skeletal conditions, and the effectiveness of treatments using FAs and HGs. From 2011 to 2019, studies predominantly addressed treatment with FAs, including both removable and fixed devices. After 2019, there was an initial exploration of the effects of skeletal anchorage for late growth stages and eruption guidance appliances for early growth stages.

### Keyword clustering

**Fig. 9:**
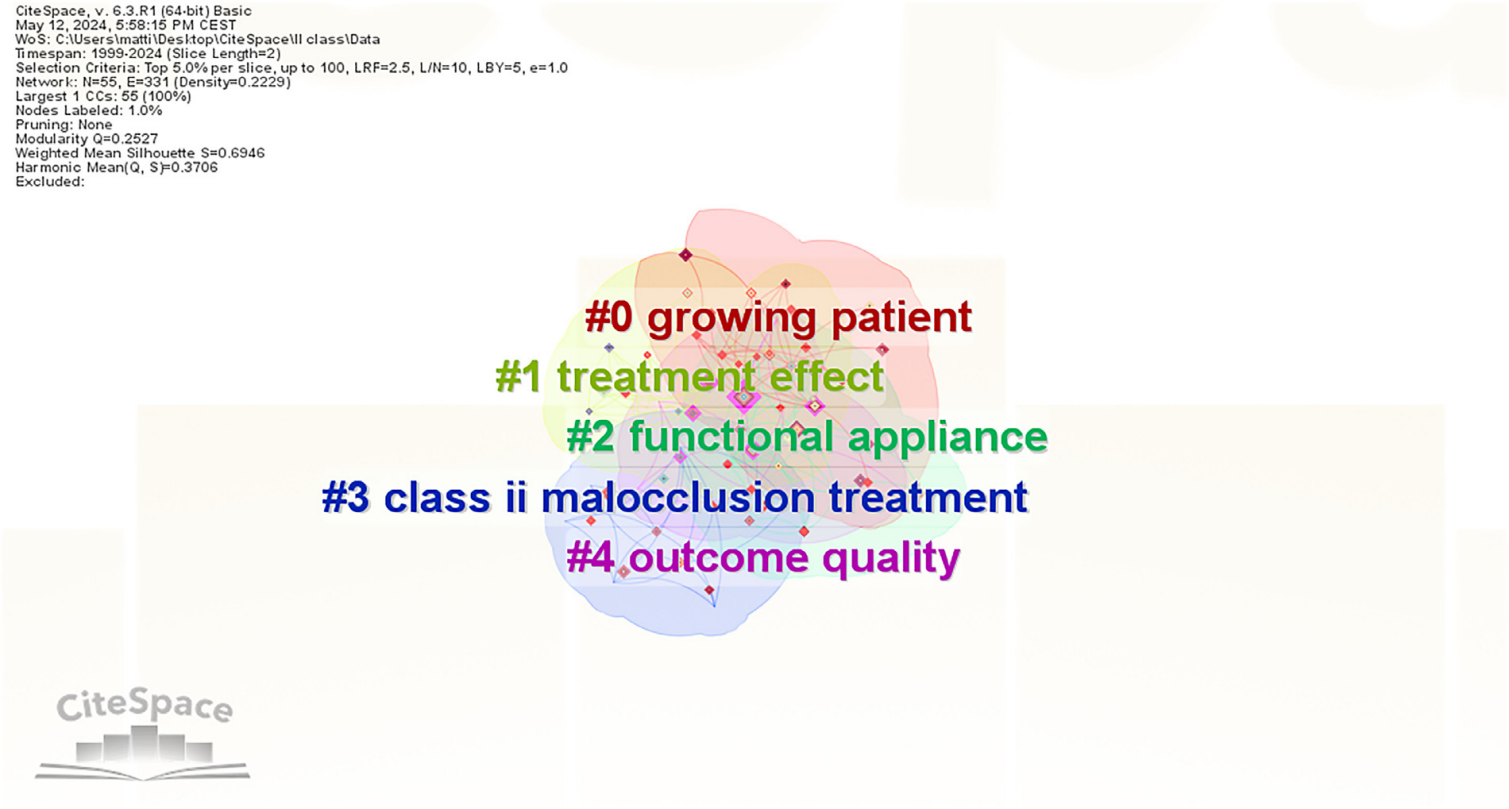
Cluster graph of keywords.

**Fig. 10:**
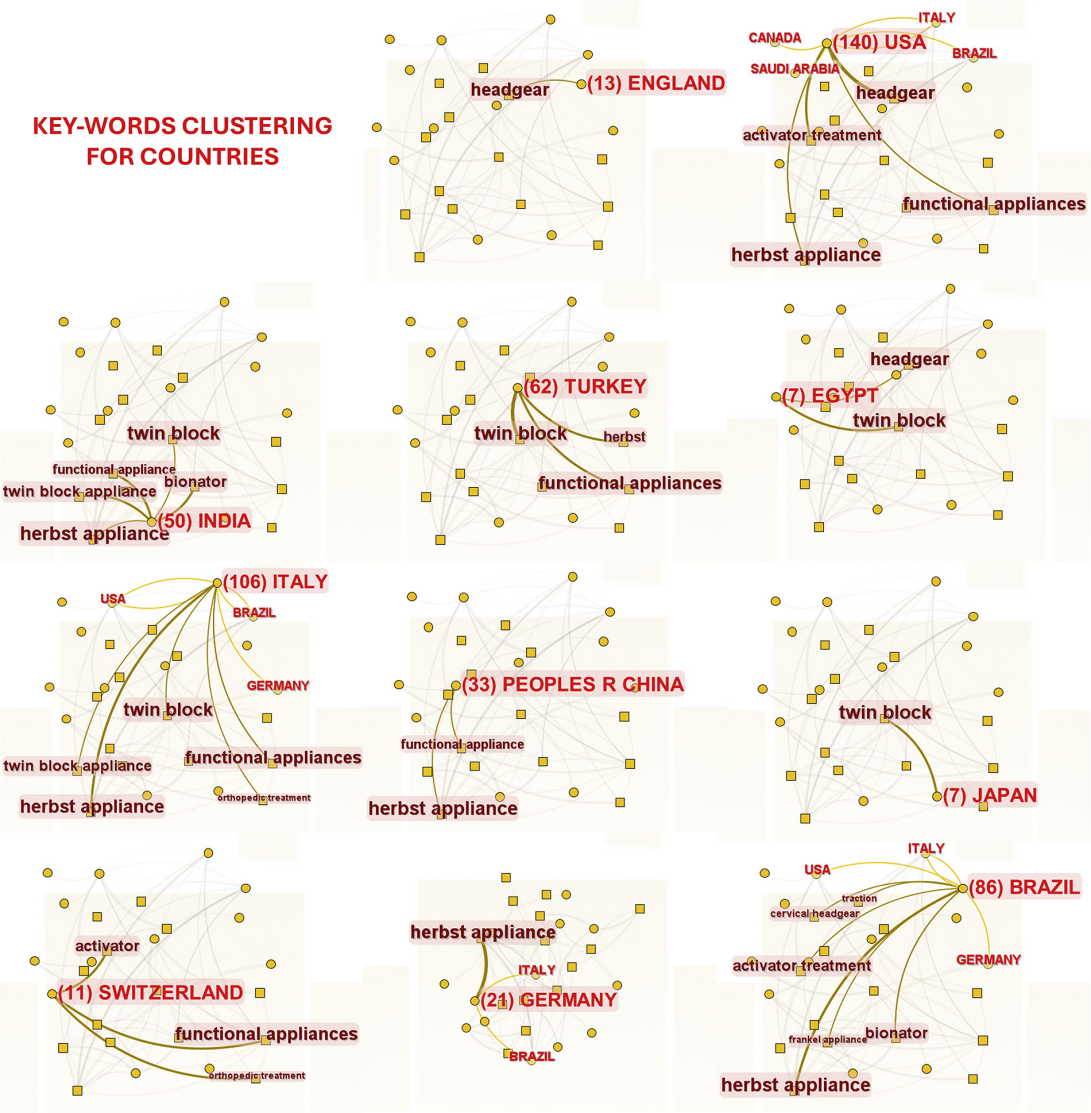
Keywords clustering for countries.

Cluster labels were derived from keywords related to the article titles, resulting in 5 distinct clusters. The clustering results, displayed in Figure 9, include “growing patient,” “treatment effect,” “functional appliance,” “Class II malocclusion treatment,” and “outcome quality.” Keywords were also clustered according to appliance types, and keyword co-occurrence was analysed in relation to countries. Regarding functional treatment, the Twin Block appliance was primarily studied in Italy and India; the Herbst appliance in Germany, Brazil, and Italy; the Bionator appliance in India and Brazil; and HG in the United States and England. Figure 10 illustrates the countries where different appliance types were investigated.

### Cited journal analysis

Table 3 lists the top 10 journals ranked by citation count, primarily from the United States, the United Kingdom, Germany, and Denmark, with the United States contributing 4 journals to the list. The *American Journal of Orthodontics and Dentofacial Orthopedics, The Angle Orthodontist*, and *European Journal of Orthodontics* occupy the top 3 positions and are recognised as authoritative journals in the field of orthodontics. The average impact factor (IF) of these journals ranges from 2.6 to 3.4 (Journal Citation Reports 2023 - jcr.clarivate.com), indicating the need for continued exploration and research in this area.

**Table 3:** Top 10 journals related to Class II malocclusion.

Ranking	Journal	Count	Impact factor	Quartile	Country	Centrality
1	*American Journal of Orthodontics and Dentofacial Orthopedics*	1123	3	Q2	US	0.31
2	*Angle Orthodontist*	782	3.4	Q2	US	0.47
3	*European Journal of Orthodontics*	768	2.6	Q3	England	0.24
4	*Seminars in Orthodontics*	280	4.2	Q1	US	0.04
5	*Journal of Orofacial Orthopedics-Fortschritte der Kieferorthopadie*	269	1.7	Q4	Germany	0.07
6	*Journal of Clinical Orthodontics*	264	0	0	US	0.05
7	*British Journal of Orthodontics*	215	0	0	England	0.09
8	*Orthodontics and Craniofacial Research*	168	3.1	Q2	Denmark	0.12
9	*Progress in Orthodontics*	156	4.8	Q1	Germany	0.06
10	*Journal of Orthodontics*	119	1.3	Q3	England	0

### Reference analysis

Table 4 presents the top 10 cited references, all of which were published between 1999 and 2021. Five studies were performed by research group from the United States, which aligns with the high publication output found for this country. The topics investigated were mainly the treatment effectiveness of FAs (removable and fixed) and HGs and the appropriate treatment timing. Two studies also addressed the topic of patients’ satisfaction and social experience. Nine studies were published between 1999 and 2007, whilst only one study (meta-analysis) can be considered recent (2021; India). The inclusion of a recent article in the top 10 list indicates sustained interest within the scientific and clinical communities in understanding the effectiveness of early Class II therapies. However, the absence of high-impact clinical studies since 2004 suggests that these earlier studies have provided a robust foundation for subsequent research, having thoroughly explored the changes and efficacy of treatment with FAs. Nonetheless, considering the analysis of citation bursts, which reveal a recent trend towards skeletal anchorage, it is reasonable to hypothesise an emerging body of evidence in the coming years that will compare the effectiveness of FAs in conjunction with miniscrews.

**Table 4:** Top 10 most cited articles related to Class II malocclusion.

Ranking/title	Year	Author/country	Citations	Journal	**Design**
1. Mandibular changes produced by functional appliances in Class II malocclusion: a systematic review	2006	Cozza Paola (Itay); McNamara James A (US)	214	*American Journal of Orthodontics and Dentofacial Orthopedics*	Review
2. Effectiveness of early orthodontic treatment with the Twin-block appliance: a multicenter, randomized, controlled trial. Part 1: dental and skeletal effects	2003	O'Brien Kevin (England); Cook Philip (US); Birnie David (Australia)	213	*American Journal of Orthodontics and Dentofacial Orthopedics*	Prospective trial
3. Treatment timing for Twin-Block therapy	2000	Baccetti Tiziano (Italy); McNamara James A (US)	187	*American Journal of Orthodontics and Dentofacial Orthopedics*	Retrospective trial
4. Outcomes in a 2-phase randomized clinical trial of early Class II treatment	2004	Tulloch J F Camilla (US)	174	*American Journal of Orthodontics and Dentofacial Orthopedics*	Prospective trial
5. Skeletally anchored Forsus Fatigue Resistant Device for correction of Class II malocclusions-a systematic review and meta-analysis	2021	Ravikumar Prasanna Arvind (India)	164	*Orthodontics & Craniofacial Research*	Review
6. Effectiveness of treatment for Class II malocclusion with the Herbst or Twin-block appliances: a randomized, controlled trial	2003	O'Brien Kevin (England); Cook Philip (US); Birnie David (Australia)	148	*American Journal of Orthodontics and Dentofacial Orthopedics*	Prospective trial
7. Treatment effects produced by the Twin-block appliance and the FR-2 appliance of Frankel compared with an untreated Class II sample	1999	Toth Laszlo Richard (Hungary); McNamara James A (US)	131	*American Journal of Orthodontics and Dentofacial Orthopedics*	Retrospective trial
8. Long-term stability of orthodontic treatment and patient satisfaction - a systematic review	2007	Bondemark Lars (Sweden); Hansen Ken (US); Pietila Terttu (Finland)	122	*Angle Orthodontics*	Review
9. Effectiveness of early treatment of Class II malocclusion	2002	Wheeler Timothy T. (US); Taylor MG (England)	105	*American Journal of Orthodontics and Dentofacial Orthopedics*	Prospective trial
10. Effectiveness of early orthodontic treatment with the Twin-block appliance: a multicenter, randomized, controlled trial. Part 2: psychosocial effects	2003	O'Brien Kevin (England); Cook Philip (US); Birnie David (Australia)	103	*American Journal of Orthodontics and Dentofacial Orthopedics*	Prospective trial

## Discussion

Bibliometric research represents a rigourous method for exploring and analysing large volumes of scientific data and is used to “weigh” the existing literature on a specific topic, providing information about research constituents, collaborations, and emerging trends.^11^^,^^12^ This information helps to define the map of existing scientific knowledge that would serve as reference for future investigation and ideas.^13^^,^^14^ In the present study, we aimed to investigate the impact of the existing scientific literature concerning the treatment of Class II malocclusion in growing patients, considering the epidemiologic relevance of this malocclusion within the orthodontic population.^15^ To achieve this objective, we utilised the Web of Science Core Collection database, which is highly regarded for scientific bibliometric analysis because of its extensive coverage of references and citations.^9^ For the visual analysis in this investigation, we employed VOSviewer bibliometric software, which provided valuable references for visual analysis in our study.^14^^,^^16^ “Headgear,” “functional appliances,” and related terms were the essential components of the search query because the aim of the study was to analyse the research on class II correction in growing individuals. In this regard, Class II malocclusion can be diagnosed mainly as mandibular retrusion, sagittal maxillary hyperplasia, or a combination of these 2 conditions, and HGs and FAs represent 2 distinct types of appliances used, respectively, to control or inhibit maxillary growth and stimulate mandibular advancement.

The volume of publications within a given time frame indicates the research development trends and the progression rate of a discipline. There has been a consistent and ongoing publication trend on the addressed topic, with a significant increase in the last decade, aligning with the classic description of scientific temporal development.^17^ The increase in publications after 2015 can be partially attributed to growing interest in skeletal anchorage and elastodontic appliances.

Keywords exhibiting citation bursts indicate periods of high citation frequency, thereby identifying research hot spots within a specific field at particular times.^18^ The emergent keywords can be detected using burst detection maps, and for the present investigation, the onset of high citation activity (Figure 9) can be divided into 3 distinct phases: before 2011, with research focussed on growth factors and skeletal conditions influencing treatment effectiveness with FAs and HGs; 2011 to 2019, with studies addressing the effectiveness of fixed FAs; and after 2019, with studies exploring emerging topics such as skeletal anchorage and eruption guidance appliances. In this regard, skeletal anchorage has been proposed and tested as a method to improve skeletal effectiveness and counteract dentoalveolar side effects related to the usage of fixed FAs, in particular incisors proclination and molar distalization. Eruption guidance appliances have reemerged in the last year in the new form of elastomeric devices that would allow the development of light, biological elastic forces. These forces would enable the correction of malocclusions during childhood, correcting the position of teeth and potentially affecting growth.^19^^,^ ^20^

The majority of literature on Class II treatment has been produced by institutions in the United States, Brazil, and Italy, particularly the Universities of Michigan, São Paulo, and Florence. Notably, many publications from the University of Florence are collaborative efforts with the University of Michigan, resulting in numerous clinical trials and literature reviews. A significant contribution to evaluating the actual effectiveness of orthopaedic-functional devices has been the use of a control group (untreated) from the American Association of Orthodontists Foundation Craniofacial Growth Legacy Collection (www.aaoflegacycollection.org). This archive consists of 9 well-known collections of longitudinal craniofacial growth records in the United States and Canada, including annual cephalograms of children who never received orthodontic treatment. Such resource is invaluable for orthodontic researchers, as it allows them to compare the effectiveness of Class II treatment against an untreated group, overcoming ethical restrictions related to recruiting untreated individuals.

Lorenzo Franchi (University of Florence, Italy), Guilherme Janson (James Cook University, Brazil), and Carlos Flores-Mir (University of Alberta, Canada) are the 3 most prolific researchers in Class II treatment for growing individuals. These researchers have predominantly focussed on evaluating the effectiveness and optimal timing of Class II fixed and removable appliances. Additionally, Guilherme Janson has concentrated on the effectiveness and treatment stability of various extraction protocols,^21^^,^^22^ whilst Carlos Flores-Mir has investigated the morphologic characteristics of patients with Class II malocclusion in relation to paediatric sleep apnoea syndrome.^23^^,^^24^ In general, the studies conducted by these authors have established a robust theoretical foundation for advancing knowledge about Class II correction in growing individuals.

Keyword clustering revealed that most studies on HG were conducted by institutions in the United States and England, whereas studies on FAs were predominantly conducted in Europe, South America, and India. This suggests that the scientific contributions of specific countries and their respective authors reflect the regional clinical trends in appliance usage. Removable FAs have not gained widespread acceptance in the United States, whereas fixed FAs attracted interest after being reintroduced by Pancherz in 1979.^25^ Notably, Germany, where the Herbst appliance was designed and invented, has the highest number of publications about this appliance. It is important to emphasise that these regional trends in the use of orthopaedic/functional devices could represent a bias that influences both the educational training of young orthodontists and the choices made by clinicians selecting the appropriate device for correction of Class II malocclusion. Moreover, this bias highlights that the efficacy of many devices tested in the literature is often limited to specific ethnic and racial groups.

From the clustering of keywords in relation to the titles, labels strictly related to the treatment of the malocclusion in question have emerged. However, no labels have emerged that suggest the use of Class II devices with other appliances or correlate them with other occlusal or orofacial characteristics. In this regard, the discussion can be focused on 2 main aspects: A) #0 growing patients and #1 treatment effects, B) #3 class II malocclusion and #4 outcome quality.

The purpose of correcting Class II malocclusion is to minimize the sagittal discrepancy between maxillary and mandibular arch. In growing individuals, this means inhibiting maxillary growth and/or stimulating mandibular growth to minimize dentoalveolar compensation, with the goal of improving function and profile aesthetic.^26^ Treatment timing is crucial to successfully correct Class II malocclusion in growing individuals. Concerning functional appliances, treatment starting at the pubertal peak was able to produce significantly greater skeletal effects (mandibular length, mandibular ramus height, advancement of the bony chin) compared with treatment started before puberty.^27^^,^^28^ At the same time, late treatment should result in minor skeletal changes and greater reliance on dental compensation.

Concerning appliance design, the main distinction for functional treatment is between fixed and removable FAs. Both modalities are effective in correcting the overjet, with few differences in skeletal cephalometric changes but with greater dentoalveolar compensation found with fixed devices (molar distalisation and lower incisor proclination).^29^^,^^30^ Concerning HGs, high-pull and cervical-pull traction are generally used to support molar distalisation and/or sagittal maxillary growth controlling molar eruption and/or vertical maxillary growth. Although HG has traditionally been associated with vertical adverse effects in terms of posterior mandibular rotation, it seems that cervical HG on average is not consistently associated with posterior rotation of the jaws or a consistent increase in vertical facial dimensions amongst patients with Class II malocclusion.^31^

When literature has tried to answer to the question of whether 2-phase treatment (early treatment with FA or HG followed by fixed appliance) is justified over 1-phase treatment,^32^ results indicated that after phase 1 of early treatment (ie, before the other group had received any intervention), there was a reduction in overjet and ANB after treatment with an FA or HG; however, when both groups had completed treatment, there was no difference in the outcomes between groups. Instead, early treatment with FAs reduced remarkably the incidence of incisal trauma compared with late treatment (30% vs 19%). As far as evidence-based orthodontics is concerned, the possibility to reduce the risk of incisor trauma is actually the only clinical indication supporting the decision to start the correction of Class II malocclusion earlier.^33^ Evidence (low to moderate) suggests that providing early orthodontic treatment for children with prominent upper front teeth is more effective in reducing the incidence of incisal trauma than providing one course of orthodontic treatment in adolescence. There appear to be no other advantages of providing early treatment when compared with late treatment.

In addition to incisor trauma, some secondary effects may justify early intervention for Class II correction. In this regard, the improvement in overjet seems to be associated with an increase in patients’ facial aesthetics, as estimated equally by dental professionals and laypeople.^34^ The latter aspect has an important relevance from the daily-life and social perspective, considering that children with skeletal Class II malocclusion are at higher risk of experiencing bullying episodes and have low self-esteem and a lower social perception. Furthermore, FAs may contribute to enlarging upper airway dimensions, specifically in the oropharyngeal region, in growing individuals with skeletal Class II malocclusion.^35^ Early FA intervention for mandibular retrognathism may help enlarge airway dimensions and decrease potential risk of obstructive sleep apnoea syndrome for growing patients in the future.^35^^,^^36^

It is likely that, in the future, current evidence regarding the effectiveness of functional orthopaedic treatment for Class II malocclusion, as well as regarding the benefit of early 2-phase treatment compared to single-phase late treatment, will be reevaluated in light of new future evidence based on the use of skeletal anchorage in growing patients.

## Limitations

The present bibliometric study used exclusively Web of Science Core Collection as a research source, as many other scientific databases such as PubMed and Embase do not provide full-text and citation analysis. Using databases that lack these elements may have led to incomplete data analysis.

Data interpretation and result visualisation could have been influenced by a certain degree of subjectivity because data interpretation requires a deep and comprehensive understanding of the topic and is not based on outcome quantification (meta-analysis).

The present bibliometric study did not analyse studies regarding postgrowth treatment approaches for Class II correction. Indeed, our study was designed to map the literature regarding Class II correction in growing individuals because this field was marked by several controversies, and different treatment philosophies are still embraced in terms of biological principles and appliance types. Further bibliometric studies could analyse scientific and clinical trends related to postgrowth therapies, such as camouflage treatment and combined orthodontic/orthognatic surgery treatments.

## Conclusions

Correction of Class II malocclusion in growing individuals using FAs or HGs has gained widespread attention because of the possibility to counteract unfavourable maxillary and mandibular growth patterns, improving skeletal disharmony and the facial attractiveness.

Keyword clustering concerning appliance types delineated a specific geographic map representing the countries with greater publication counts on FAs and HGs. Because the geographic publication trend could reflect regional clinical trends in appliance usage, ethnic and racial bias may represent a limitation of the actual scientific evidence.

Controversy still exists over whether to perform single-phase or 2-phase treatment, with the primary rationale for early intervention being the mitigation of incisal trauma risk alongside aesthetic improvement.

## Conflict of interest

None disclosed.
